# Influence of Biofilm Maturity on the Antibacterial Efficacy of Cold Atmospheric Plasma in Oral Microcosm Biofilms

**DOI:** 10.3390/biomedicines12051056

**Published:** 2024-05-10

**Authors:** Hee-Eun Kim

**Affiliations:** Department of Dental Hygiene, Gachon University College of Medical Science, Incheon 21936, Republic of Korea; hekim@gachon.ac.kr; Tel.: +82-32-820-4375

**Keywords:** antibacterial, biofilm, extracellular polysaccharide, pathogenicity, plasma

## Abstract

As biofilms mature, biomass and extracellular polysaccharide (EPS) content increases, enhancing pathogenicity. Therefore, this study aimed to evaluate the antibacterial efficacy of cold atmospheric plasma (CAP) against oral microcosm biofilms and the influence of biofilm maturity on treatment. Oral microcosm biofilms were cultured on hydroxyapatite disks for 2 and 6 days. Based on the treatment and biofilm maturity, these were subsequently allocated into six groups (N = 19 each): Groups 1 and 2 were incubated with distilled water for 1 min; Groups 3 and 4 were treated with CAP for 2 min, and Groups 5 and 6 were treated with 0.12% chlorhexidine gluconate for 1 min. Groups 1, 3, and 5 represent 2-day biofilms, and Groups 2, 4, and 6 represent 6-day biofilms. Treatments were repeated daily for 5 days. Antibacterial efficacy was analyzed by measuring oral biofilms’ red fluorescence intensity (Ratio_R/G_) and quantifying EPS content and bacterial viability. The Ratio_R/G_ was 1.089-fold and 1.104-fold higher in Groups 4 and 6 than in Groups 3 and 5 following antibacterial treatment, respectively (*p* < 0.001). EPS content increased by 1.71-fold in Group 6 than in Group 5 (*p* < 0.001). Bacterial survival rate was the lowest in Group 3 (*p* = 0.005). These findings underscore the relevance of CAP treatment in maintaining antibacterial efficacy regardless of the biofilm development stage, highlighting its potential utility in oral care.

## 1. Introduction

Oral biofilms are complex, structured communities of diverse bacteria that adhere to intraoral substrates [1], creating a competitive environment that controls the overgrowth of individual bacterial species and maintains homeostasis in the oral cavity [2]. However, various environmental factors that affect the host, such as poor oral hygiene, frequent carbohydrate consumption, and stress, can disrupt the homeostasis of oral biofilms. These disturbances in biofilm ecology can lead to dysbiosis, increasing the risk of oral diseases such as dental caries and periodontal diseases [2,3]. Hence, managing and controlling the ecological system of oral biofilms are crucial for preventing and treating oral diseases.

In the field of dentistry, plasma technology is recommended for controlling oral biofilms [4]. Plasma, a partially ionized gas with neutral atoms, molecules, ions, and electrons, exhibits strong antimicrobial capabilities, facilitating precise and targeted use. This specificity is particularly advantageous for targeting oral biofilms at clinical sites. CAP’s antibacterial potential largely stems from its ability to produce reactive species, including reactive oxygen and nitrogen species (RONS), that can impair and neutralize microorganisms [5,6,7]. Previous studies have reported the effects of a nitrogen-based CAP jet on the morphology of *Streptococcus mutans* using transmission electron microscopy [6,8]. Generally, a low pressure or a high temperature is required for plasma generation [7]. However, cold atmospheric plasma (CAP) can be generated at atmospheric pressure and room temperature; this property distinguishes it from conventional plasma [9]. Thus, CAP can be safely applied to the oral cavity, ensuring no damage to the intraoral tissues. The CAP jet is engineered to emit plasma while maintaining a defined distance from the tissues. A study showed that, after 2 or 10 min of treatment, bacteria suffered structural damage and cytoplasmic release due to RONS production [10].

Bacterial characteristics can markedly influence the antibacterial efficacy of CAP [11]. This aspect is crucial, particularly because most previous studies have focused on single bacterial strains or biofilms comprising a single species [11]. For instance, a previous study demonstrated the complete sterilization of *Streptococcus* and *Lactobacillus* biofilms using an argon (Ar)-based CAP brush for 60 s [12]. Nevertheless, considering the complexity of the microecosystem of oral biofilms, which includes approximately 1000 bacterial species, applying CAP to limited species biofilm models may not fully reflect clinical settings [13,14]. Therefore, addressing these factors is essential for ensuring clinically relevant and effective use of CAP in complex oral microbial environments.

The oral microcosm biofilm model, constructed using human salivary inoculants, is an accurate representation of the complex and metabolically diverse interactions among various bacterial species present in the oral environment [15]. It also provides an observable platform for forming extracellular polysaccharides (EPSs), primary virulence factors associated with oral diseases [16]. As the oral biofilm matures, a biomass increase characterized by EPS deposition occurs, thereby increasing biofilm pathogenicity [17]. EPSs not only promote bacterial cell adhesion and growth but also encourage cross-kingdom interactions, inducing a shift toward the predominance of pathogenic species [18]. Most critically, EPSs impede the penetration of external agents, particularly antimicrobials, acting as a protective barrier [19]. This pronounced defensive role of EPSs implies that an increase in their content can decrease bacterial susceptibility to antimicrobial treatments [17]. However, the influence of biofilm maturity on the efficacy of CAP jet treatment remains unexplored. Therefore, the aim of this study was to evaluate the antibacterial efficacy of CAP against oral microcosm biofilms and determine whether this efficacy is influenced by biofilm maturity. We hypothesized that antibacterial treatment type and oral microcosm biofilm maturity do not significantly interact (H_0_).

## 2. Materials and Methods

### 2.1. Ethical Considerations

This study was conducted with the approval of the Institutional Review Board of our institution (approval number: 1044396-202107-HR-165-01) and was conducted in accordance with the Declaration of Helsinki. Participants were recruited after a briefing on the research purpose and provision of the methods, following which informed consent was obtained. Saliva samples were collected from donors who signed consent forms.

### 2.2. Sample Size Determination and Specimen Preparation

The required sample size for a two-way analysis of variance (ANOVA) across the six groups was calculated using G*Power (version 3.1; Heinrich-Heine-University Düsseldorf, Düsseldorf, Germany). The total sample size was calculated to be 111 for an effect size (f) of 0.3, a probability of alpha error of 0.05, and a power of 0.80.

In total, 114 hydroxyapatite (HA) disks (Himed, Old Bethpage, NY, USA) with a 7-mm diameter and 2-mm height were prepared. The disks were subjected to a stepwise polishing process with abrasive papers ranging from 800–1200 grit, followed by refinement using a polishing device (Model M-Prep 5; Allied High Tech Products, Inc., Compton, CA, USA). In alignment with previously detailed methods [20], each HA disk was placed in an acrylic mold, ensuring 1 mm clearance atop the disk to facilitate biofilm development. The specimens were sterilized with ethylene oxide gas.

### 2.3. Formation of Oral Microcosm Biofilms

Saliva was collected from healthy individuals (*n* = 3, pooled saliva, aged between 20 and 30 years, non-smokers) with no dental decay or gum disease, and those who had not taken antibiotics in the last 3 months. These individuals were asked to avoid all oral hygiene activities for 24 h before donating their saliva. The collected saliva was filtered using sterilized glass wool following stimulation (Duksan Chemicals, Ansan, Republic of Korea). This filtered saliva (1.5 mL) was applied to HA disk specimens arranged in 24-well cell culture plates (SPL Life Sciences, Pocheon, Republic of Korea). The plates were incubated at 37 °C in an atmosphere with 10% CO_2_ (BB15 CO_2_ incubator; Thermo Fisher Scientific, Waltham, MA, USA) for 4 h. Post incubation, the saliva was removed from the HA disk specimens, and a fresh medium (final pH 7.0) containing 0.5% sucrose (0.1 mL) and basal medium mucin (1.4 mL) was added to each specimen. This medium was subsequently replaced daily for either 2 or 6 days, allowing for the development of oral microcosm biofilms under the same temperature and CO_2_ conditions. According to the findings of a preliminary experiment regarding the effect of biofilm thickness on the penetration of QLF-D’s blue light, biofilm thickness was adjusted to 1 mm, which did not impede light penetration.

### 2.4. Group Allocation and Treatment

**Figure 1:**
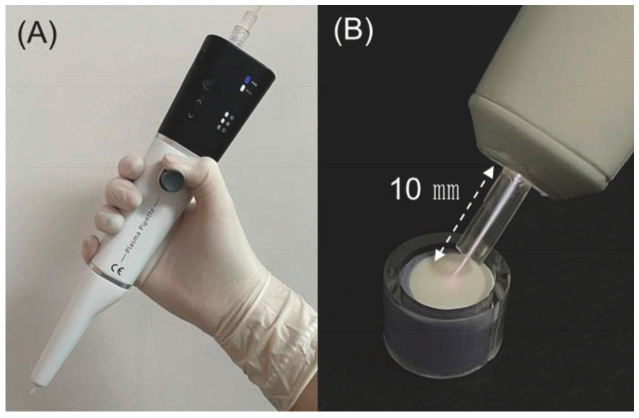
Argon-based plasma pipette. (**A**) Body of the pipette. (**B**) Emitted plasma stream with the white dotted line measuring 10 mm, the precise length of the plasma exposure from the nozzle tip to the surface of the oral microcosm biofilm [21].

Oral microcosm biofilms were allocated into six groups based on the biofilms’ treatment and maturity: Groups 1 and 2 were treated with 1.5 mL of distilled water (DW) for 1 min, serving as the negative control for 2-day and 6-day biofilms, respectively. Groups 3 and 4 received CAP treatment with an Ar-gas-based plasma pipette (Femto Science, Hwaseong, Republic of Korea) operated at a power setting of 4 W, a voltage of 10 kV, and a frequency of 100 kHz (Figure 1A) [21]. CAP treatment was administered for 2 min, and the output pressure of the regulator was adjusted to 0.02 MPa; the plasma column temperature was maintained at 40 °C, and a consistent distance of 10 mm was maintained between the top of the handpiece nozzle and the biofilm surface (Figure 1B). Group 3 represented 2-day biofilms, while Group 4 corresponded to 6-day biofilms. Groups 5 and 6 were subjected to 1-min treatment with 1.5 mL of 0.12% chlorhexidine gluconate solution (CHX; hexamedine, Bukwang Pharm Co., Ltd., Ansan, Republic of Korea), serving as the positive control for 2-day and 6-day biofilms, respectively. Following each treatment, all biofilms were washed thrice with 1.5 mL of phosphate-buffered saline (PBS) to remove any remaining antibacterial substances. Once all treatments were completed, 1.5 mL of fresh medium was replenished. All biofilms were incubated at 37 °C in an atmosphere containing 10% CO_2_ after treatment. This process was repeated daily for 5 days.

### 2.5. Analysis of Red Fluorescence Intensity: Ratio_R/G_

**Figure 2:**
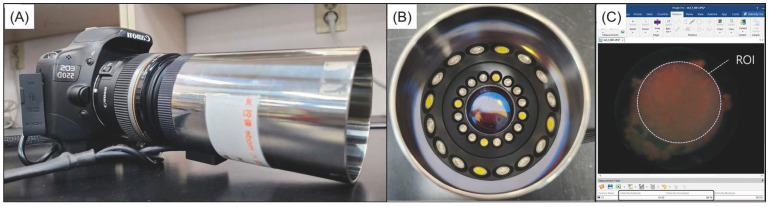
Digital setup for quantitative analysis using quantitative light-induced fluorescence-digital (QLF-D) camera. (**A**) Body of the digital fluorescence camera. (**B**) The illumination system of QLF-D camera. (**C**) The target area for examination (region of interest, ROI) is shown on a fluorescence snapshot of the oral microcosm biofilm utilized to measure the red and green fluorescence intensities [23].

Before and after antibacterial treatment, biofilms were imaged using blue light from a quantitative light-induced fluorescence-digital (QLF-D) camera (QLF-D Biluminator™2+; Inspektor Research Systems BV, Amsterdam, Netherlands) (Figure 2A,B). The imaging conditions were set to a shutter speed of 1/60 s, an aperture value of 7.1, and an ISO speed of 1600. The gap between the lens and biofilm was constant at 10 cm, while biofilms’ fluorescence intensity was recorded at an ambient temperature. Red fluorescence intensity in these images was analyzed using Image PRO (version 11; Media Cybernetics, Inc., Silver Spring, MD, USA). For each biofilm image, a region of interest of the same size was used to analyze red and green intensities (Figure 2C). Subsequently, RatioR/G, which is the ratio of red to green intensity, was calculated; a higher value indicates greater biofilm pathogenicity [22].

### 2.6. Analysis of EPS Content Using Phenol-Sulfuric Acid Colorimetric Assay

After the 5-day treatment regimen, HA disks laden with biofilm were washed thrice with 1.5 mL of PBS to clear any unattached bacteria. Subsequently, these disks were placed into conical tubes containing 1 mL PBS. Specimens were vortexed for biofilm detachment (VM-96A; Lab Companion, Seoul, Republic of Korea) and subjected to ultrasonic agitation (SHB-1025; Saehan Sonic, Seoul, Republic of Korea) for 1 min, resulting in the formation of a biofilm suspension. Next, 50 µL of this suspension was placed into a 96-well microtiter plate, followed by the addition of 150 µL of concentrated sulfuric acid to initiate the reaction. Next, 30 µL of a 5% phenol solution (by weight) was added, and the samples were incubated at 90 °C for 5 min in a water bath for complete reaction. After cooling to room temperature, the absorbance of the colored samples was measured at 490 nm using a spectrophotometer (CM-5; Minolta, Tokyo, Japan). Higher absorbance values indicate higher concentrations of EPS within the biofilm [24].

### 2.7. Bacterial Viability: Colony Count and Bacterial Survival Rate

As previously described, the prepared biofilm suspensions were serially diluted (10^−1^ to 10^−6^), after which 100 µL of each diluted suspension was plated onto tryptic soy agar plates (Becton Dickinson and Co., Franklin Lakes, NJ, USA) containing 5% sheep blood (final pH 7.0). After 72 h of incubation, the bacterial colony counts were determined and expressed as log CFUs/mL.

A LIVE/DEAD BacLight Bacterial Viability Kit (LIVE/DEAD kit; Invitrogen^TM^, Waltham, MA, USA) was used to assess the bacterial survival rate. SYTO-9 and propidium iodide were mixed at a 1:1 ratio, as per the guidelines of the manufacturer. Following the initial preparation, 3 µL of the premixed staining solution was added to 1 mL of the biofilm suspension. This mixture was incubated at 37 °C in the dark for 15 min. Subsequently, 5 µL of this suspension was placed onto a microscopic slide and covered with a glass cover slip. The stained biofilm suspension was imaged at 100× magnification using a confocal laser scanning microscope (Zeiss LSM 700; Carl Zeiss AG, Oberkochen, Germany) at dual excitation wavelengths, 488 nm and 555 nm. Image acquisition and processing were performed using ZEN 2009 (Carl Zeiss AG). Image PRO was utilized to evaluate the intensities of green and red fluorescence intensities throughout the captured image area. The proportion of the area stained green (Ratio_G/G+R_) was computed, representing the bacterial survival rate; a higher value indicates a greater bacterial survival rate [25].

### 2.8. Statistical Analysis

IBM SPSS Statistics version 28.0 (SPSS Inc., Chicago, IL, USA) was employed to analyze all collected data, with statistical significance set at *p* < 0.05. Normality tests were conducted for all the outcomes. To assess the effect of the interaction between treatment type and biofilm maturity on Ratio_R/G_ and EPS amount, a two-way ANOVA was conducted. Subsequently, an independent *t*-test was conducted to compare the differences in Ratio_R/G_ and EPS amounts according to biofilm maturity for each treatment agent, with significance levels adjusted using the Bonferroni method. Additionally, to compare bacterial counts among groups, one-way ANOVA followed by Tukey’s post-hoc analysis was performed. To compare bacterial survival rates (Ratio_G/G+R_), a Kruskal–Wallis test was conducted, with subsequent post-hoc analysis using the Mann–Whitney *U* test and significance levels adjusted using the Bonferroni correction.

## 3. Results

### 3.1. Pathogenicity of Oral Biofilms

**Figure 3:**
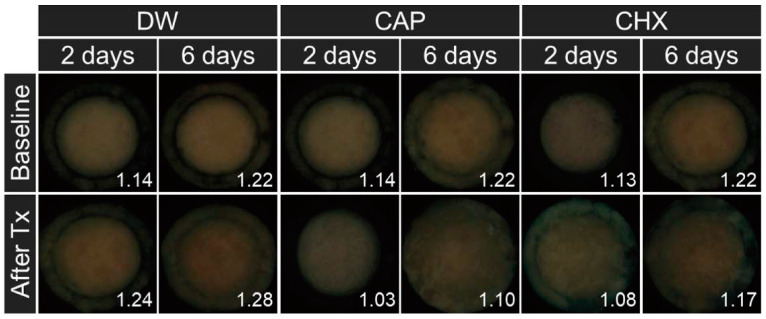
Changes in the red fluorescence of oral microcosm biofilms following antibacterial treatment. The number at the bottom right of the image represents the Ratio_R/G_ of the oral microcosm biofilm. CAP, cold atmospheric plasma; CHX, chlorhexidine; DW, distilled water; Tx, treatment.

Biofilm pathogenicity, measured based on Ratio_R/G_, revealed a significant interaction between the type of antibacterial treatment and biofilm maturity (*p* = 0.002, Table 1). Specifically, Ratio_R/G_ was notably higher in the 6-day than in the 2-day biofilms, indicating an increase in pathogenicity with biofilm age. This effect was observed post-CAP and CHX treatment, wherein Ratio_R/G_ was 1.089-fold and 1.104-fold higher in 6-day biofilms (Groups 4 and 6), respectively, than in the 2-day biofilms (Groups 3 and 5) (adjusted *p* < 0.001, Table 1, Figure 3).

### 3.2. EPS Content in Oral Microcosm Biofilms

**Figure 4:**
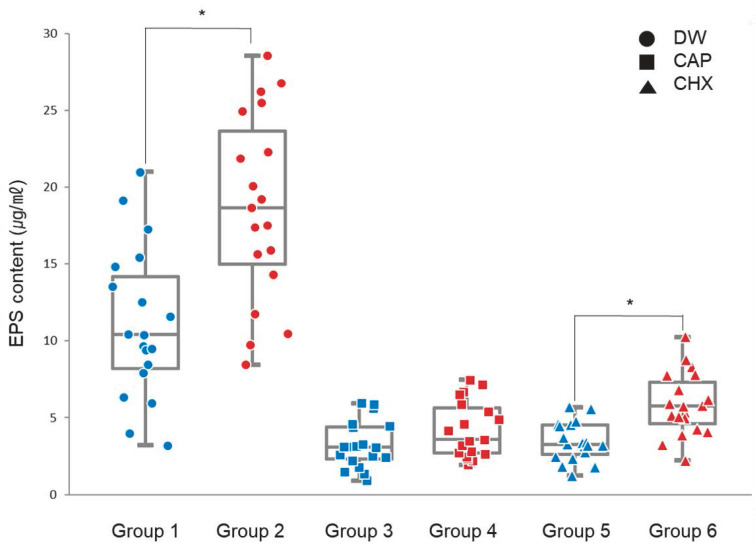
Amount of extracellular polysaccharides in the oral microcosm biofilms according to antibacterial treatment and biofilm maturity. Blue represents 2-day biofilms and red indicates 6-day biofilms. * Adjusted *p*-values were obtained from independent *t*-tests applied as post-hoc analyses following a 2-way ANOVA. CAP, cold atmospheric plasma; CHX, chlorhexidine; DW, distilled water; EPS, extracellular polysaccharides.

There was an interaction between the treatment type and biofilm maturity (*p* < 0.001). In CAP-treated biofilms (Groups 3 and 4), the EPS content in the 6-day biofilms (4.21 ± 1.83) was 1.30-fold greater than those in the 2-day biofilms (3.25 ± 1.52); however, this difference was not significant (adjusted *p* = 0.85, Figure 4). Nevertheless, biofilms treated with DW (Groups 1 and 2) or CHX (Groups 5 and 6) resulted in a significant increase in EPS content in 6-day biofilms (18.71 ± 6.10; 5.87 ±2.03), which were 1.69- and 1.71-fold higher, respectively, than that in 2-day biofilms (11.08 ± 4.88; 3.44 ± 1.26; adjusted *p* < 0.001, Figure 4).

### 3.3. Bacterial Viability

**Figure 5:**
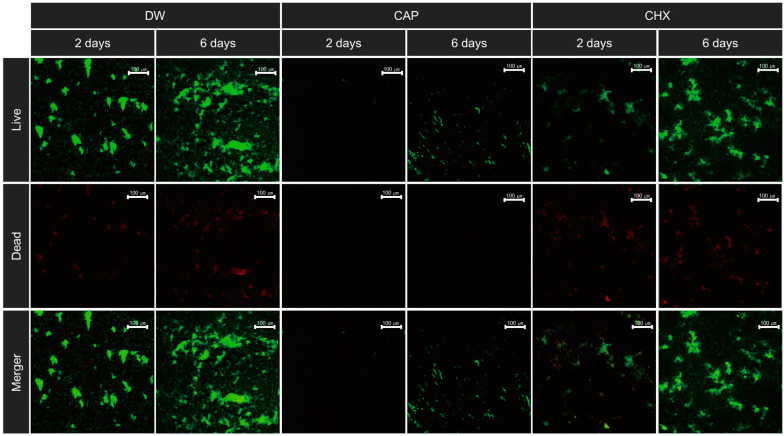
Representative confocal laser scanning micrographs (100× magnification) of bacteria stained with live (green) and dead (red) markers. CAP stands for cold atmospheric plasma; CHX stands for chlorhexidine; DW stands for distilled water.

No antibacterial treatment type and biofilm maturity interaction effect was observed on bacterial viability. However, biofilms treated with CAP (Groups 3 and 4) or CHX (Groups 5 and 6) exhibited significantly lower bacterial counts than those treated with DW (Groups 1 and 2) (*p* < 0.001, Table 2); however, no significant differences between the 2-day and 6-day biofilms in terms of biofilm maturity were observed (Table 2). Notably, the lowest bacterial survival rate was found in the 2-day biofilms treated with CAP (Group 3), highlighting the efficacy of CAP in the early stages of biofilm development (*p* = 0.005; Table 2). Additionally, CAP treatment was associated with a noticeable reduction in bacterial aggregation (Figure 5).

## 4. Discussion

In this study, we investigated how oral biofilm maturity interacts with antibacterial treatment type to influence treatment efficacy against oral microcosm biofilms, considering oral biofilm pathogenicity, EPS amount, and bacterial viability. Oral microcosm biofilm pathogenicity was analyzed using QLF-D technology. Blue light (405 nm) emitted by the QLF-D camera excites red extrinsic fluorophores present in bacterial metabolites, such as porphyrins, and possibly extrinsic and intrinsic polysaccharides [26,27]. This excitation results in the emission of red or orange fluorescence [27,28]. As biofilms mature, porphyrins and EPSs accumulate, leading to a higher intensity of red fluorescence [29]; this suggests that higher red fluorescence intensity correlates with increased biofilm pathogenicity [30]. Our findings indicated that the Ratio_R/G_ of 6-day biofilms was higher than that of 2-day biofilms after treatment with CAP or CHX. A previous study reported that biofilm maturity correlates with an increase in red fluorescence intensity [22]. The accumulation of biomass, including EPSs, is augmented in biofilms of advanced maturity. Given that the rate of solute penetration inversely correlates with biomass density, the diffusion of antibacterial agents is inhibited in aged biofilms [31,32]. This inverse relationship may result in reduced antibacterial efficacy against older biofilms. This could be the reason for the relatively higher Ratio_R/G_ observed in the 6-day biofilms following antibacterial treatment, a phenomenon that was particularly pronounced following CHX treatments.

In the present study, EPS accumulation within the biofilm after antibacterial treatment was analyzed. Figure 4 shows a significant increase in EPS amount in 6-day biofilms compared with that in 2-day biofilms in the DW-treated groups. This suggests that, as the biofilm ages, the accumulation of EPSs within it increases. A similar pattern was observed in the CHX-treated groups despite the established antibacterial efficacy of CHX. The significantly greater amount of EPSs in 6-day biofilms than in 2-day biofilms suggests decreased bacterial susceptibility to CHX treatment in more mature biofilms [17]. This phenomenon could be attributed to the ability of EPSs to create a microenvironment that restricts CHX [33,34,35,36], possibly due to the negative charge of EPSs, which may interfere with the movement of the positively charged CHX [33,37]. Additionally, EPSs can form a protective barrier around the bacteria [35,38]. However, CAP treatment did not result in significant differences in the amount of EPSs between 2- and 6-day biofilms, suggesting that CAP consistently inhibits EPS production across various stages of biofilm development. Furthermore, our findings imply that CAP disrupts the internal structure of older biofilms more effectively than CHX, enhancing its potential to compromise the integrity of these more established biofilms [39]. This observation was substantiated by bacterial viability (Table 2). Although bacterial cell counts did not significantly differ between CAP and CHX treatments, the bacterial survival rates were lowest in 2-day biofilms treated with CAP among the groups. This demonstrated the heightened efficacy of CAP in reducing bacterial survival in the earlier stages of biofilm development.

Our findings highlighted that younger biofilms were more vulnerable to antibacterial treatment, as evidenced by pathogenicity assessments, whereas CAP treatment appeared to be beneficial, even for eradicating more mature biofilms, as concluded from EPS amount quantification. Based on previous findings that CAP induces oxidation to destabilize amyloid-beta peptide aggregation [40], theorizing that CAP treatment could similarly disrupt EPS aggregation in biofilms is plausible. Although this previous study did not explicitly examine biofilms, the shared characteristics of peptide and EPS aggregation suggest that the oxidizing effects of CAP may impede EPS cohesiveness within biofilm structures. Additionally, based on the reported concentrations of oxidizing agents, such as H_2_O_2_ at the vacuum-water interface and within the water bulk during plasma generation [41], plasma-generated substances can permeate into liquids. These findings suggest that the reactive oxygen species (ROS) produced by CAP can be delivered to biofilms, potentially destabilizing EPS structure and causing cellular destruction [42]. This implies that CAP may effectively disrupt biofilm integrity, as substantiated by the confocal laser scanning microscope images depicted in Figure 5. These images provide visual confirmation of the theoretical capability of CAP to compromise biofilm architecture, suggesting that even established biofilms can be effectively targeted by CAP-based antibacterial treatments.

Despite these findings, this study had some limitations. First, the potential cytotoxic effects of CAP treatment in the oral cavity, particularly concerning the concentration of ROS generated, could not be tested. While previous studies have indicated the non-toxic nature of plasma-generated reactive species due to their short-lived nature [12], the optimal concentration of ROS that balances therapeutic efficacy without inducing cytotoxicity remains to be elucidated [6]. Future studies should establish a safe and effective range for ROS concentrations in CAP treatments, for better applicability in clinical settings. Second, differences in the treatment duration between CAP and CHX in our study highlight the need for standardizing treatment protocols. In this study, the duration of CAP treatment was set to 2 min, considering the diameter of the plasma stream, which was roughly 1 mm, directed at a disk of 7 mm, and the output pressure was maintained at 0.02 MPa. CHX treatment was conducted for 1 min, adhering to the guidelines provided by the manufacturer. Therefore, future research should explore the effects of varying treatment durations and conditions for CAP to identify the most effective and practical application method in oral healthcare. Third, the substrates used to cultivate oral microcosm biofilms were the HA disks, which, while serving as an adequate model, differ from natural human enamel in several aspects. However, we used the same protocol to prepare the surfaces of the HA disks as that for human enamel, thus minimizing any potential effects on biofilm formation [43]. Future studies can replicate these experiments in human enamel substrates to more closely mimic in vivo conditions and assess CAP’s treatment efficacy in a more clinically relevant context. Given the notable effect of CAP treatment in compromising the structure of biofilms and reducing EPS levels, forthcoming studies can delve into the fundamental mechanisms driving these phenomena. How CAP-induced ROS interact with key biofilm constituents, including EPS, is expected to shed light on the intricate molecular dynamics at play in the disruption of biofilms by CAP. The potential applications of CAP treatment extend beyond treating oral biofilms to possibly addressing other biofilm-associated infections, particularly those unresponsive to traditional treatments. CAP’s ability to undermine biofilm cohesion and diminish bacterial survival rates highlights its potential relevance across various medical contexts, meriting further investigative efforts. Finally, extending beyond the oral cavity, CAP’s treatment applicability should be explored in other biofilm-related infections, especially those resistant to conventional treatments. CAP’s ability to undermine biofilm cohesion and diminish bacterial survival rates highlights its potential relevance across various medical contexts, meriting further investigative efforts. By addressing these limitations of the study, the understanding of CAP’s role in biofilm management and its contribution to the development of effective and safe treatment modalities for biofilm-related diseases in dentistry and beyond are expected.

## 5. Conclusions

This study provides novel insights into the relationship between antibacterial treatments and biofilm maturity. The results show that mature biofilms exhibit lower susceptibility to treatment than do younger biofilms. However, the use of CAP treatment shows promise in effectively addressing the problem of mature biofilm formation by leveraging oxidants to penetrate and disrupt EPS structures, thereby reducing biofilm pathogenicity. CAP is potentially a better alternative to traditional antimicrobials, such as CHX, as its efficacy is consistent across different biofilm stages and may have fewer side effects. These findings underscore the potential of CAP in oral care and warrant further investigation to optimize its application and ascertain its safety profile.

## Figures and Tables

**Table 1:** Ratio_R/G_ of oral biofilms according to antibacterial treatment and biofilm maturity.

Groups	Treatments	Maturity (Days)	N	Baseline	After Tx.
1	DW	2	19	1.15 (0.12)	1.21 (0.05)
2		6	19	1.22 (0.04)	1.25 (0.02)
Adjusted *p*-values ^†^					0.274
3	CAP	2	19	1.14 (0.09)	1.01 (0.05)
4		6	19	1.23 (0.06)	1.10 (0.04)
Adjusted *p*-values ^†^					<0.001
5	CHX	2	19	1.14 (0.11)	1.05 (0.05)
6		6	19	1.23 (0.05)	1.16 (0.05)
Adjusted *p*-values ^†^					<0.001

Data are expressed as the mean (standard deviation). ^†^ Adjusted *p*-values were calculated using independent *t*-tests and post-hoc analyses after a 2-way ANOVA. CAP, cold atmospheric plasma; CHX, chlorhexidine; DW, distilled water; EPS, Extracellular polysaccharide; Tx, treatment.

**Table 2:** Bacterial viability after antibacterial treatment.

Groups	Treatments	Maturity (Days)	Colony Count (log CFUs/mL)	Bacterial Survival Rate (Ratio_G/G+R_)
1	DW	2	7.19 (0.52) ^a^	0.92 (0.88–0.94) ^a^
2		6	7.48 (0.24) ^a^	0.91 (0.89–0.94) ^a^
3	CAP	2	5.96 (0.66) ^b^	0.69 (0.62–0.75) ^b^
4		6	6.38 (0.39) ^b^	0.73 (0.61–0.84) ^ab^
5	CHX	2	6.15 (0.60) ^b^	0.73 (0.68–0.74) ^ab^
6		6	6.28 (0.45) ^b^	0.76 (0.69–0.82) ^ab^
		*p*-values	<0.001 ^†^	0.005 ^‡^

All data are expressed as the mean (standard deviation) or median (interquartile range). ^†^ The *p*-value was calculated using a one-way ANOVA, followed by Tukey’s post-hoc test. ^‡^ The *p*-value was calculated using the Kruskal-Wallis test followed by post-hoc Mann-Whitney *U* tests with Bonferroni correction. ^a,b^ Significant differences between groups are denoted by distinct superscript letters within the same column. Analyses were conducted on 19 specimens per group for bacterial counts and on 9 specimens per group for bacterial survival rate. CAP, cold atmospheric plasma; CFUs, colony-forming units; CHX, chlorhexidine; DW, distilled water.

## Data Availability

The data presented in this study are available on request from the corresponding author. The data are not publicly available due to intellectual property rights restrictions.
